# Medical haematology trial eligibility and the Duffy null‐associated neutrophil count: A cross‐sectional study

**DOI:** 10.1002/hem3.70236

**Published:** 2025-10-06

**Authors:** Olga Tsiamita, Laura Aiken, Mohsin Badat, Gill Lowe, Funmi Oyesanya, Sonia Wolf, Lauren E. Merz, Andrew Hantel, Stephen P. Hibbs

**Affiliations:** ^1^ Barts Health NHS Trust London UK; ^2^ Blizard Institute Queen Mary University of London London UK; ^3^ Department of Cardiovascular Sciences University of Birmingham Birmingham UK; ^4^ NHS Blood and Transplant London UK; ^5^ Department of Medical Oncology Dana‐Farber Cancer Institute Boston Massachusetts USA; ^6^ Department of Medicine, Mass General Brigham Division of Haematology/Oncology Boston Massachusetts USA; ^7^ Center for Bioethics Harvard Medical School Boston Massachusetts USA; ^8^ Wolfson Institute of Population Health Queen Mary University of London London UK

The Duffy null variant is caused by a single‐nucleotide polymorphism in the promoter region of the *ACKR1* gene, which encodes atypical chemokine receptor 1, also called the Duffy antigen receptor for chemokines (DARC). This variant leads to an absence of Duffy antigens on red blood cells. Due to impacts on neutrophil chemotaxis, the variant is also associated with a 30%–40% lower circulating neutrophil count but no increased risks of infection or decreased stress response.^1^ One US cohort found 10% of Duffy null individuals had an absolute neutrophil count (ANC) less than 1.5 × 10^9^/L.^2^ The same study reported a Duffy null‐associated neutrophil count (DANC) reference interval of 1210 to 5390/μL,^2^ which has recently been validated in an international cohort.^3^ This phenomenon of DANC is also known as ACKR1/DARC‐associated neutropenia (ADAN).^4^

People of all genetic ancestries can have the Duffy null phenotype, but the variant is mostly prevalent in individuals of African and Middle Eastern ancestry as it confers partial protection against *Plasmodium vivax* which is endemic in those regions.^5^ For example, amongst individuals self‐identifying as Black in the United States (US) and the United Kingdom (UK), approximately 65% and 80% are Duffy null, respectively,^6^ and prevalence amongst Saudi Arabian nationals within two different provinces of Saudi Arabia is around 50%–80%.^7^, ^8^

ANC criteria are often incorporated into clinical trial eligibility criteria, but these criteria rarely take Duffy status into account. Thus, patients with DANC may be disproportionately excluded from cancer trials due to their lower baseline ANC. A recent cross‐sectional study assessed 289 phase 3 clinical trials for the five most common cancer types, reporting that 77% of trials excluded individuals for ANC values that would be within the normal range for someone with the Duffy null variant.^9^

We hypothesised that restrictions excluding Duffy null individuals could also be present in clinical trials within medical haematology. Medical haematology (previously called “non‐malignant” or “benign” haematology) encompasses many chronic disorders that disproportionately affect populations from minoritised ethnic groups^10^ who are likely to have a higher prevalence of the Duffy null variant.

To address this hypothesis, we designed a cross‐sectional study with the aim of assessing the proportion of medical haematology clinical trials with exclusion criteria for ANC values within the DANC reference range. We aimed to assess both explicit exclusions (i.e., studies requiring ANC above a value within the normal range for DANC) and implicit exclusions (i.e., studies requiring ANC within normal limits, commonly defined on reference intervals established on Duffy non‐null individuals). The study followed the Strengthening the Reporting of Observational Studies in Epidemiology (STROBE) reporting guidelines. As only publicly available clinical trial data were used, the study did not require research ethics committee review.

We sought to assess trials for conditions representing major areas within medical haematology. As there are no comprehensive lists of conditions within these subspecialties, we selected the following common or significant conditions and identified related search terms as further outlined in the Supporting Information Methods: bone marrow failure disorders (aplastic anaemia, bone marrow failure and paroxysmal nocturnal haemoglobinuria), haemoglobinopathy (sickle cell disease and thalassaemia), haemostasis and thrombosis disorders (antiphospholipid syndrome, haemophilia A, haemophilia B, inherited platelet disorders, inherited thrombophilia, venous thrombosis and von Willebrand disease) and immune haematology (autoimmune haemolytic anaemia, immune thrombocytopenia and thrombotic thrombocytopenia).

We searched studies registered on ClinicalTrials.gov with trial start dates between October 1, 2022 to October 1, 2024. In addition to the search terms described above, we restricted the search to adult, interventional, phase 2/3 trials. The database was queried on October 21, 2024. The data extraction method, definition of exclusion criteria and screening method followed published methodology^9^ and is further described in the Supporting Information S1: Methods. Data were screened by two individuals (O.T. and L.A.) and discrepancies were resolved by a third checker (S.H.) (interrater reliability *κ* = 0.98).

Our search returned 202 studies, of which 33 were excluded as noninterventional studies or with a treatment indication outside of our predefined conditions of interest. The remaining 169 studies involved an investigational medical product (IMP) and were grouped into the four mutually exclusive broad therapeutic fields of bone marrow failure disorders (*n* = 32), haemoglobinopathies (*n* = 33), haemostasis and thrombosis (*n *= 55), and immune haematology (*n* = 49); these studies formed the analytical cohort (Figure 1).

**Figure 1:**
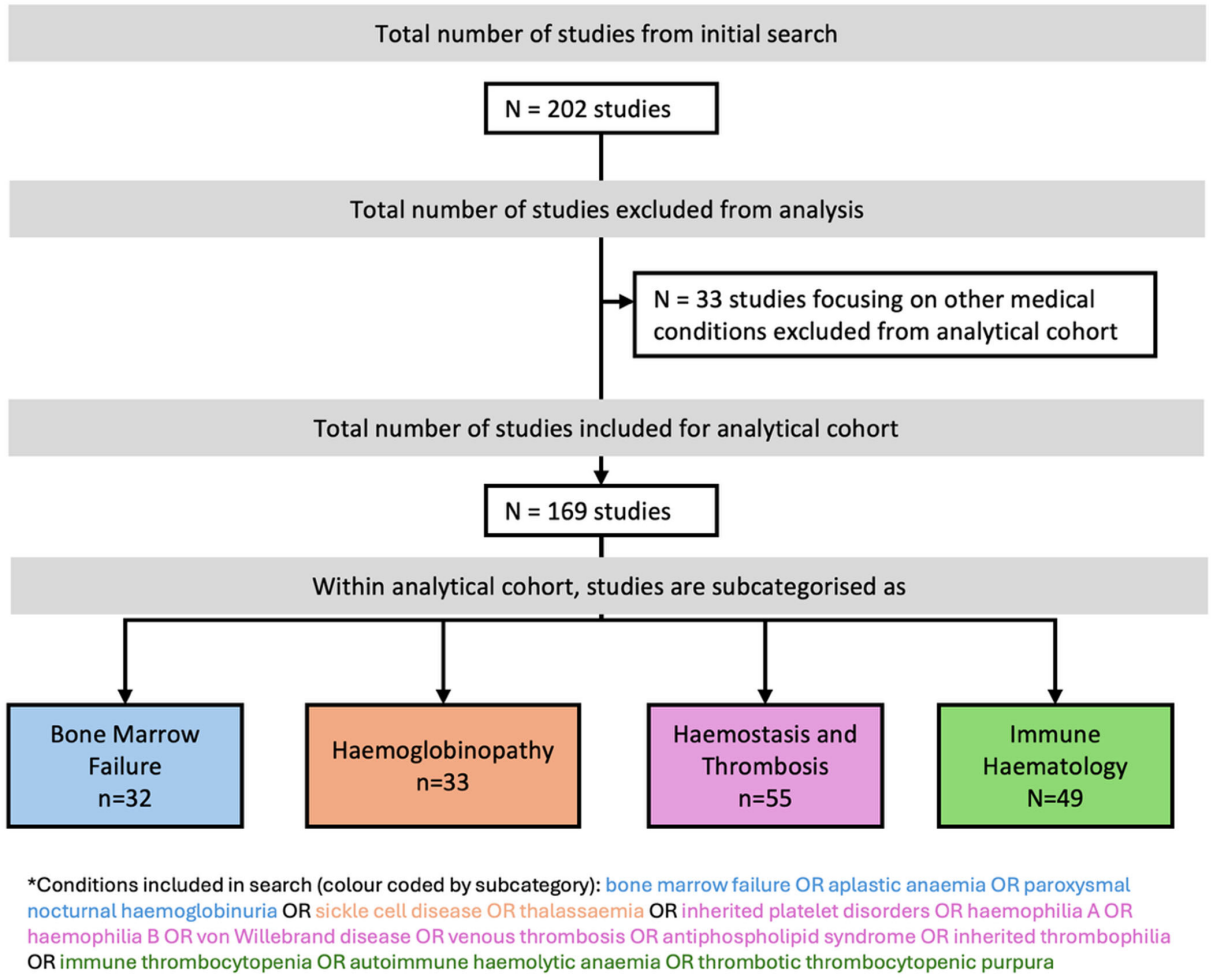
**Flow diagram of search and screening results**.

Within our analytical cohort, eligibility criteria which potentially excluded individuals with ANC values within the Duffy null normal range were identified in 17 trials (10.1%). Of these, 7 trials (4.1%) included explicit exclusions, and 10 trials (5.9%) had implicit exclusions (Figure 2). Trials with explicit exclusions typically required an ANC greater than 1.5 × 10^9^/L while those with implicit exclusions required either the absence of laboratory abnormalities, absence of cytopenias, or total white blood cell count (WBC) over a specified threshold. We did not observe any exclusions for trials investigating bone marrow failure disorders, which reflects the diagnostic *requirement* of a low ANC for many of these disorders. If bone marrow failure disorder trials are excluded (*n* = 32), the total frequency of exclusions across our cohort is 12.4%.

**Figure 2:**
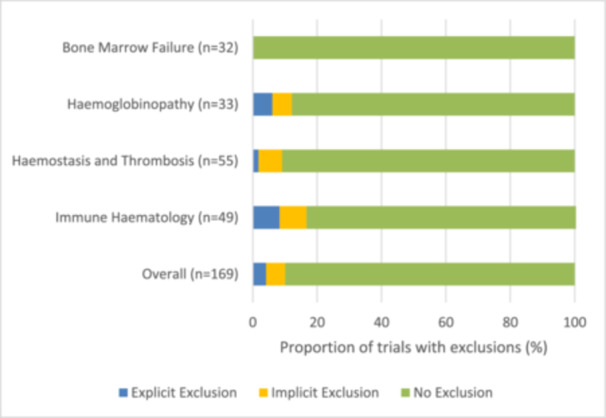
**Proportion of medical haematology clinical trials that excluded patients for ANC within the Duffy null‐associated neutrophil count reference interval by disease group.** The number of trials included in each disease group is included in parentheses.

We determined the type of investigational medicinal product (IMP) studied in each of the 17 trials containing explicit or implicit exclusions. Of these, 10/17 (59%) investigated immune modulators (e.g., monoclonal antibodies directed at B‐lymphocytes, calcineurin inhibitors), 2/17 (12%) investigated gene therapy products, and 2/17 (12%) investigated small molecules inducing haemoglobin F. The remaining three studies investigated a thrombopoietin analogue, an iron chelator, and a bi‐specific antibody targeting factor VII and platelets.

We assessed estimated or actual numbers of enroled participants for each trial within the analytical cohort. Trials with explicit or implicit exclusions included fewer participants (mean = 81 participants) than the average trial size across the whole cohort (mean = 117 participants). We did not observe any suggested eligibility modifications for individuals with the Duffy null variant.

Our estimate of 10.1% is likely to be an underestimate of the true number of clinical trials that exclude patients for ANC values within the DANC normal range. Where full trial protocols were available, we assessed them directly. However, in many cases, only trial registry summaries were accessible, which may omit exclusion criteria present in full protocols. Additionally, relevant exclusions may take place on an individual basis after discussions with sponsors, and we were not able to account for these. Furthermore, nine trials (5.3%) required ANC over 1.0 × 10^9^/L (*n* = 7) or over 1.2 × 10^9^/L (*n* = 2). For the purposes of this study, these were classified as not excluding within the DANC range. However, recent data suggest that healthy individuals with DANC may have neutrophil counts as low as 0.5 × 10^9^/L.^3^, ^11^

The exclusions we identify are not trivial. First, they limit the potential participant pool for clinical trials. For example, among trials studying treatments for sickle cell disease (SCD), 4/21 (19.0%) used eligibility criteria that could exclude within the DANC normal range. Given that a significant proportion of individuals with SCD are also Duffy null (e.g., 75% in a US cohort^12^), many of whom will have lower ANC, these eligibility criteria risk impeding trial recruitment.

Second, these criteria disproportionately affect individuals of African and Middle Eastern ancestry, in whom the Duffy null variant is common. This may contribute to unrepresentative trial cohorts, as is documented in haemophilia clinical trials.^13^ Under‐representation in clinical trials can have downstream effects on treatment outcomes. For instance, 7 of 34 trials (20.6%) in immune thrombocytopenia (ITP) included restrictive ANC criteria, potentially hampering efforts to address recognised ethnic disparities in this condition.^14^

The overall rate of ANC‐related exclusions in medical haematology trials (10.1%) is significantly lower than that reported in solid tumour oncology trials (76.5%).^9^ In oncology, ANC restrictions may be used with the intent of reducing the risk of neutropenic sepsis during cytotoxic chemotherapy. In our analysis of medical haematology trials, many trials with ANC‐related exclusions investigated drugs with a known risk of neutropenia (e.g., anti‐CD38 antibodies). For example, immune haematology trials frequently test agents with immune suppressive (though rarely myelosuppressive) mechanisms of action, which may explain the higher frequency of restrictive ANC criteria in this disease group. However, some trials with ANC‐related exclusions evaluated treatments such as a thrombopoietin receptor agonist, for which there is no clear rationale of affecting neutrophil counts or immune function. These patterns were also seen in oncology as restrictive ANC criteria were frequently applied in trials involving only hormonal therapies, suggesting a broader issue of over‐reliance on common ANC thresholds.^9^

There were no explicit or implicit ANC exclusion criteria within the DANC reference range for bone marrow failure syndrome trials. On the contrary, inclusion in these trials often required a low ANC such as less than 0.5 × 10^9^/L, agnostic to Duffy status. It is unclear how or if this impacts people with the Duffy null variant. Of note, the Camitta criteria include ANC <1.5 × 10^9^/L as a diagnostic criterion for aplastic anaemia, which raises the possibility of overdiagnosis in those with the Duffy null variant.^15^ Further research is necessary to understand if current ANC thresholds used for diagnosis, risk stratification, and response assessment in bone marrow failure disorders should be re‐evaluated based on Duffy status.

There are several strengths of our study. To our knowledge, this is the first systematic assessment of ANC‐related exclusion criteria in medical haematology trials. We applied a reproducible search strategy across multiple disease areas and performed independent data screening with high inter‐rater reliability. Limitations include likely underestimation due to reliance on registry summaries where full protocols were unavailable; inability to capture informal sponsor‐level decisions; and conservative classification of trials requiring ANC ≥ 1.0–1.2 × 10^9^/L as nonexcluding, despite evidence that some healthy Duffy null individuals may fall below this range. Further research should directly evaluate the real‐world impact of such criteria on screening outcomes for Duffy null individuals.

In summary, approximately 10% of phase 2 and phase 3 interventional trials in medical haematology employ eligibility criteria that are likely to disproportionately exclude individuals with the Duffy null variant. These criteria should be critically appraised for clinical necessity, and removed where safe to do so. If ANC or related parameters are considered necessary for a particular trial, Duffy‐adjusted thresholds should be considered.^16^ To improve inclusivity, recruitment efficiency, and the generalisability of trial findings, clinical trial designs must account for natural variation in ANC associated with Duffy status.

## AUTHOR CONTRIBUTIONS

**Olga Tsiamita**: Conceptualization; investigation; methodology; writing—review and editing; writing—original draft; project administration; formal analysis. **Laura Aiken**: Investigation; validation; writing—review and editing. **Mohsin Badat**: Writing—review and editing. **Gill Lowe**: Writing—review and editing. **Funmi Oyesanya**: Writing—review and editing. **Sonia Wolf**: Writing—review and editing. **Lauren E. Merz**: Writing—review and editing; methodology. **Andrew Hantel**: Methodology; writing—review and editing. **Stephen P. Hibbs:** Conceptualization; methodology; supervision; writing—review and editing; writing—original draft; validation; visualization; formal analysis.

## CONFLICT OF INTEREST STATEMENT

O.T. reports receiving honoraria from Sobi, travel support from Hemab and spousal employment in MSD. L.A. reports receiving travel support from CSL Behring, Roche, and Takeda UK. G.L. has received research fellow funding from Biomarin and honoraria for educational session delivery in the last 2 years from Amgen, AbbVie, Sobi, Sanofi and CSL Behring. L.E.M. reports receiving personal fees from 23 and Me and J&J (consultancy), and is a Scientific Editor for *HemaSphere*. A.H. reports receiving personal fees from AstraZeneca, BMS, GSK and AbbVie (advisory boards), Indy Haematology Review and the American Journal of Managed Care (speaker's bureau), Jazz and Genentech (consultancy) and Real Chemistry (spousal employment). S.P.H. is a Scientific Editor for *HemaSphere*. For the remaining authors, no relevant conflicts of interest were declared.

## ETHICS STATEMENT

As only publicly available clinical trial data were used, the study did not require research ethics committee review.

## FUNDING

LEM is supported by a grant from the Doris Duke Foundation. AH is supported by the National Cancer Institute of the National Institutes of Health under Award Number K08 CA273043 and the Conquer Cancer Foundation of the American Society of Clinical Oncology Career Development Award. SPH is supported by a HARP doctoral research fellowship, funded by the Wellcome Trust (Grant number 223 500/Z/21/Z).

## Supporting information

Supporting methods final.

## Data Availability

The data that support the findings of this study are available from the corresponding author upon reasonable request. The extracted data are available upon reasonable request to the corresponding author.
