# Supplementary material for: Medical haematology trial eligibility and the Duffy null‐associated neutrophil count: A cross‐sectional study
Source: Hemasphere. 2025 Oct 6;9(10):e70236. doi: 10.1002/hem3.70236 (PMC12498218; doi:10.1002/hem3.70236)
Supplement: Supplementary file 1 — Supporting methods final. [file HEM3-9-e70236-s001.docx]

**Supplementary Methods**

**Search, screening, and data extraction procedures**

These procedures are amended from methodology used by Hibbs et al^1^ exploring ANC restrictions in solid cancer clinical trials.

**Database selection**: clinicaltrials.gov was selected as the database of choice. US regulations mandate registration and publication of US clinical trial records on this site, UK regulations mandate registration and publication of UK clinical trials on this site as one of two options, it captures a large number of global clinical trials, and it supplies adequate data fields for selecting trials of interest.

**Search**: The following searches were performed using the categories and options available on clinicaltrials.gov. If a category or option is not mentioned, it was not included in the search. Separate searches were performed for each condition/disease term listed. Searches were not limited by geography. All searches were performed on 21 October 2024.

*Condition/Disease (as a single search with OR function between conditions):*

- - bone marrow failure
  - aplastic anaemia
  - paroxysmal nocturnal haemoglobinuria
  - sickle cell disease
  - thalassaemia
  - inherited platelet disorders
  - haemophilia A
  - haemophilia B
  - von Willebrand disease
  - venous thrombosis
  - antiphospholipid syndrome
  - inherited thrombophilia
  - immune thrombocytopenia
  - autoimmune haemolytic anaemia
  - thrombotic thrombocytopenic purpura

*Participant Age* Adult (18 - 64) OR Older adult (65+)

*Study Phase*: II OR III

*Study Type*: Interventional

*Study Start*: From: 1 October 2022 To: 1 October 2024

**Screening**: Search results were manually screened to exclude trials that did not test interventions to treat medical haematology conditions (listed above). Studies were grouped into the following mutually exclusive categories based on the type of condition treated.

a. Bone Marrow Failure Disorders: bone marrow failure, aplastic anaemia, or paroxysmal nocturnal haemoglobinuria

b. Haemoglobinopathies: sickle cell disease, or thalassaemia

c. Haemostasis and Thrombosis: inherited platelet disorders, haemophilia A, haemophilia B, von Willebrand disease, venous thrombosis, antiphospholipid syndrome, inherited thrombophilia

d. Immune Haematology: immune thrombocytopenia, autoimmune haemolytic anaemia, thrombotic thrombocytopenic purpura

**Data Extraction**: For studies included for further review, corresponding clinicaltrials.gov records were reviewed, and eligibility criteria extracted verbatim. If a full trial protocol was attached to the registry entry, the trial protocol was reviewed. Criteria were categorised into the mutually exclusive categories outlined in the Definitions below, with verbatim criteria extracted for any difficult categorisations. Each study was screened and extracted independently by two reviewers. Discrepancies were resolved by a third reviewer.

**Definitions of exclusions**

1. Explicit exclusion: report of neutrophil count (quantitative) within Duffy null reference range in the study exclusion criteria.
2. Implicit exclusion: report of broader lab parameter (qualitative) within Duffy null normal range implied in the study exclusion criteria.
3. No exclusion (not A or B)

**eTable 1**: Data extraction template

Adapted from Supplementary Methods in Hibbs et al^1^

| **Source of data element** | **Data element** | **Description of data element** | **Notes** |
| --- | --- | --- | --- |
| Clinicaltrials.gov | NCT Number | Clinical trial registration number |  |
|  | Study Title | Title of Study |  |
|  | Study URL | Link to clinicaltrials.gov study record |  |
|  | Brief Summary | Description of trial |  |
|  | Conditions | Condition/disease treated |  |
|  | Interventions | Drugs, procedures, devices, or biological agents being tested |  |
|  | Sponsor | Name of trial sponsor |  |
|  | Phases | Trial phase (II, II/III, or III) |  |
|  | Funder Type | Primary trial funder: NIH, Other Government, Industry, Other |  |
|  | Study Type | Confirmed to ensure study is listed as interventional |  |
|  | Locations | Sites at which study is being performed |  |
|  | Estimated enrolment | Total number of participants a study aimed to enrol |  |
|  | Actual enrolment | Total number of participants that were enrolled on a study |  |
| Study team | Haematology Subspecialty | Categorisation of condition type into broad disease categories: Bone Marrow Failure, Haemoglobinopathy, Haemostasis and Thrombosis, Immune Haematology |  |
|  | Medical Haematology Trial | Yes/No | This row was aligned across reviewers; discrepancies were then catalogued and a third reviewer gave a final determination. |
|  | Harmonised Include/Exclude Decision | Include/Exclude | This row reports the final determination made from the consensus conference above. |
|  | Full protocol available | Yes/No | These rows were aligned across reviewers; discrepancies were then catalogued and reviewed by a third reviewer to make a final determination |
|  | Any neutrophil-related criteria present | Yes/No |  |
|  | Explicit neutrophil count-based criteria present | Yes/No |  |
|  | Free text comments on neutrophil-related criteria | Describe neutrophil criteria |  |
|  | Harmonised neutrophil category | No restriction, implicit restriction, explicit restriction | These rows report the final determination made from the consensus conference above. |
|  | Additional details on harmonised neutrophil category | Any additional neutrophil criteria description needed |  |

**References**

1. Hibbs SP, Aiken L, Vora K, et al. Cancer Trial Eligibility and Therapy Modifications for Individuals With Duffy Null–Associated Neutrophil Count. *JAMA Network Open*. 2024;7(9):e2432475. doi:10.1001/jamanetworkopen.2024.32475
